# Usability Evaluation of Digital Health Applications for Older People With Depressive Disorders: Prospective Observational Study in a Mixed Methods Design

**DOI:** 10.2196/66271

**Published:** 2025-11-28

**Authors:** Magdalini Chatsatrian, Katharina Kunde, Jennifer Bosompem, Jan Dieris-Hirche, Nina Timmesfeld, Rainer Wirth, Georg Juckel, Magdalena Pape, Anna Mai, Chantal Giehl, Bianca Ueberberg, Horst Christian Vollmar, Ina Carola Otte, Theresa Sophie Busse

**Affiliations:** 1 Institute of General Practice and Family Medicine, Faculty of Medicine, Ruhr University Bochum, Bochum, Germany; 2 Department of Psychosomatic Medicine and Psychotherapy, LWL-University Hospital Bochum, Ruhr University Bochum, Bochum, Germany; 3 Department of Medical Informatics, Biometry and Epidemiology, Ruhr University Bochum, Bochum, Germany; 4 Department of Geriatric Medicine, University Hospital Marien Hospital Herne, Ruhr University Bochum, Bochum, Germany; 5 Department of Psychiatry and Psychotherapy, LWL-University Hospital Bochum, Ruhr University Bochum, Bochum, Germany; 6 Department of Clinical Psychology and Psychotherapy, University of Bamberg, Bamberg, Germany; 7 Digital Health, Department of Health, Faculty of Human Medicine, Witten/Herdecke University, Alfred-Herrhausen-Str. 50, Witten, 58455, Germany, 49 2302926 ext 708

**Keywords:** depression, depressive, usability, digital health, eHEALS, health literacy, mHealth, mobile health, mental health, app, applications, gerontology, geriatric, old, older, elder, elderly, aging, aged

## Abstract

**Background:**

Digital health applications (DiGA) have been integrated into Germany’s health care system since 2019, offering certified medical devices for various health conditions. This study focuses on deprexis and Selfapy, the first 2 permanently approved DiGA for depressive disorders in Germany, to evaluate their usability for people ≥60 years. The study’s significance is underscored by the underrepresentation of older people in previous DiGA studies, accompanied by an emergent risk of inequalities in distribution for this vulnerable population.

**Objective:**

This study assessed the usability of DiGA deprexis and Selfapy for adults aged ≥60 years with mild to moderate depression. The more user-friendly option will be chosen for the DiGA4Aged project’s upcoming randomized controlled trial.

**Methods:**

The prospective observational study uses the People at the Centre of Mobile Application Development (PACMAD) usability model in a mixed methods design. The study’s multistage data collection encompasses sociodemographic data and quantitative questionnaires about health literacy (European Health Literacy Survey Questionnaire [HLS-EU-Q16]), electronic health literacy (revised German eHealth Literacy Scale [GR-eHEALS]), media affinity, depressive symptoms (9-item Patient Health Questionnaire [PHQ-9]), and perceived usability (System Usability Scale [SUS]), as well as a qualitative think-aloud and semistructured interview. Participants were equally allocated to use either deprexis or Selfapy. Recruitment of 18 participants was conducted at 3 hospital departments (ie, psychiatry, psychosomatics, and geriatrics) in spring 2024. Participants were eligible if they were aged ≥60 years, were diagnosed with mild or moderate depressive disorder, owned a digital device, and gave written consent to participate.

**Results:**

Quantitative analysis revealed age, gender, depressive severity, and health literacy parity between both groups. Selfapy users displayed marginally lower technical proficiency and lower usability scores. Qualitative data showed lower usability among participants in the Selfapy group due to design-related errors and higher cognitive load. Despite visual, psychomotor, and cognitive challenges, participants endorsed both DiGA for older users, stressing the importance of assistance and practicing the usage.

**Conclusions:**

Reported difficulties in usability may help to improve future DiGA development for older people, especially as the willingness to use DiGA exists.

## Introduction

Depressive disorders represent an increasingly urgent public health issue, having already been identified by the World Health Organization as the condition most likely to rank second in the disability-adjusted life years index by 2030 [1]. Depressive disorders are associated with a significant reduction in quality of life and performance, along with major economic consequences [2].

Between 2001 and 2020, the point prevalence of self-reported depressive symptoms stood at 34% worldwide [3]. A survey was conducted across Europe to determine the prevalence of depressive symptoms within the past 2 weeks (assessed via self-report using the 8-item Patient Health Questionnaire [PHQ-8]). The result was a prevalence of 6.6% for the European Union as a whole (men 5.2% and women 7.9%), whereas in terms of age-standardized prevalence, people aged ≥65 years and above (9%) were most affected in the European Union. In Germany, a comparatively higher prevalence of 9.2% was recorded (women 10.8% and men 7.6%). People in the 15 to 29 years age group in Germany were particularly affected (11%), whereas approximately 7% of individuals aged ≥65 years showed signs of depressive symptoms [4].

There are already considerable shortcomings in treatment in Germany [5]. In particular, the demand for psychotherapy is still insufficiently met, leading to long waiting times before treatment begins [6].

Internet-based interventions may be one way of providing low-threshold access to treatment. While more than 10,000 smartphone apps are available for mental or behavioral health, evaluating these apps to ensure privacy protection, usability, and interoperability is a major challenge [7].

In Germany, efforts are underway to precisely ensure this evaluation through a standardization process, thereby giving patients direct access to quality-assured applications. Therefore, digital health applications (DiGA or Digitale Gesundheitsanwendungen) have been integrated into Germany’s health care system since the German Digital Healthcare Act came into effect in 2019. DiGA, offered as apps and web-based applications, are certified as low-risk medical devices that can be prescribed by physicians and psychotherapists or dispensed after approval by health insurance companies [8]. A prescription is possible after successfully passing an assessment procedure of the Federal Institute for Drugs and Medical Devices. This is accompanied by the entry in the DiGA directory either as temporarily approved DiGA for 1 year pending reassessment or as permanently approved [9]. The approval by the Federal Institute for Drugs and Medical Devices requires evidence of the so-called positive care effect, meaning that the DiGA has been proven to contribute either to an improved management of the disease or to enhanced patient health [10].

Currently, 26 of the overall 56 listed DiGA address indications in mental health, making this category the largest of 12. To this date (July 5, 2024), 4 DiGA (ie, deprexis, edupression.com, Novego, and Selfapy) are permanently listed for use in depressive disorders, and another 3 DiGA are temporarily approved for 1 year (elona therapy Depression, MindDoc auf Rezept, and My7steps App) [11].

The first 2 permanently approved DiGA for treating depressive disorders were deprexis and Selfapy [11]. While these DiGA offered patients an opportunity to take advantage of treatment options for depressive symptoms, not all patients could be reached through this channel. The usage behaviors of certain age groups differ according to a report on the implementation and development of DiGA between 2020 and 2022. During the period under review, deprexis was prescribed approximately 10,000 times, with the frequency of use decreasing steadily among those aged ≥65 years. During the same period, Selfapy was prescribed about 9000 times in total, with almost no users in the age group >60 years [12]. Moreover, the approval studies of both deprexis [13,14] and Selfapy [15] did not include participants aged >65 years.

These findings illustrate the necessity of incorporating accessibility and participation considerations for the older population because depressive disorders are among the most common mental illnesses in older people [16]. Approximately 4% to 5% of individuals aged 65 to 79 years in Germany are affected by depressive symptoms [17]. Owing to the global trend of an aging population [18], the prevalence in this population group will steadily increase. Although, as mentioned earlier, other age groups in Germany are more severely affected by depressive symptoms, it is crucial to ensure that DiGA, as a state-supported concept, are beneficial and accessible to all individuals and that no one is excluded from this growing path of digital health care.

While expanding digital health care services such as DiGA could address these deficits, at the same time, inequalities in distribution might be reinforced, especially for vulnerable groups such as older people [19]. To ensure they can also benefit from such offers, the usability of DiGA should meet the needs of this user group. Various studies (eg, [20,21]) have shown that older people face specific challenges when using mobile apps: visual, psychomotor, and cognitive limitations due to aging and disease can impair usability. For instance, font size, visual contrast, small screens, and complex menu navigation pose considerable obstacles to use [20,21].

This study aimed to evaluate the usability of the DiGA deprexis and Selfapy for people aged ≥60 years with mild or moderate depressive disorders. The evaluation is based on the People at the Centre of Mobile Application Development (PACMAD) usability model [22]. The more user-friendly DiGA will be selected for the following randomized controlled trial (RCT) in the overall project DiGA4Aged.

## Methods

### Study Design and Participants

This prospective observational study adopted a convergent mixed methods design, involving the combination of both quantitative and qualitative data to obtain a more comprehensive understanding of the research topic. The design was stated as concurrent, with greater emphasis placed on qualitative data and triangulation of data occurring on the level of interpretation. Both quantitative and qualitative data were collected at a single time point (single phase) [23].

Owing to the recent integration of DiGA into Germany’s health care system since 2019, hardly any evidence was available before this study’s conception in 2022. We searched the database of the Federal Institute for Drugs and Medical Devices for approval studies of the DiGA manufacturers and reports about the prescription and usage of DiGA. Furthermore, we searched PubMed for articles published from database inception to October 1, 2022, using the keywords (“depression” OR “depressive disorder” OR “mental health”) AND (“DiGA” OR “health app” OR “eHealth” OR “mobile app” OR “smartphone app”) without language restrictions. We identified systematic reviews on mobile apps with regard to mental health; however, none of them specifically included DiGA. Even now, the body of evidence on DiGA is only growing slowly.

The recruitment and assessment periods took place from February to April 2024 in 3 hospital departments: psychiatry, psychosomatics (both from Landwirtschaftsverband Westfalen-Lippe–University Hospital Bochum), and geriatrics (Marien Hospital Herne). In an endeavor to recruit a heterogeneous sample, the purposive selection by physicians and researchers at these departments was based on sociodemographic characteristics, health, and perceived electronic literacy, as well as perceived media affinity, alongside the eligibility criteria (Textbox 1). No distinction was made in terms of gender. In each department, the first 3 participants included in the study used Selfapy, followed by the next 3 using deprexis.

Textbox 1. **Eligibility criteria for participation.**

**Inclusion criteria**

- Aged ≥60 years
- Diagnosis of mild or moderate depressive disorder
- Ownership of a digital device suitable for digital health applications (DiGA) use
- Written consent to participate

**Exclusion criteria**

- Acute psychotic symptoms
- Acute suicidal tendencies or self-endangerment that results in an immediate need for treatment
- Severe intellectual impairment
- Motor and sensory impairments that prevent the use of a digital device
- Insufficient knowledge of the German language
- Advanced and incurable disease
- Diagnosis of bipolar disorder or schizophrenia
- Current participation in another intervention study
- Current use of a DiGA for the treatment of depressive disorder

### Ethical Considerations

The study received ethical approval from the Ethics Committee of the Ruhr University Bochum (23‐7901). The reporting adhered to the GRAMMS (Good Reporting of a Mixed Methods Study) guideline for mixed methods studies in health services research [24] and to the STROBE (Strengthening the Reporting of Observational Studies in Epidemiology) statement [25]. Participants were compensated with an expense allowance of €25 (US $29.24) per hour for their participation in the study. All participants were informed about the objectives of the study and the possible risks. They gave their written consent and were able to ask questions on site. Participation was voluntary, and they could withdraw at any time without suffering any disadvantages. None of the participants took advantage of this option. After the survey, the data were pseudonymized. All data was anonymized for the manuscript.

### Procedures

**Figure 1.**
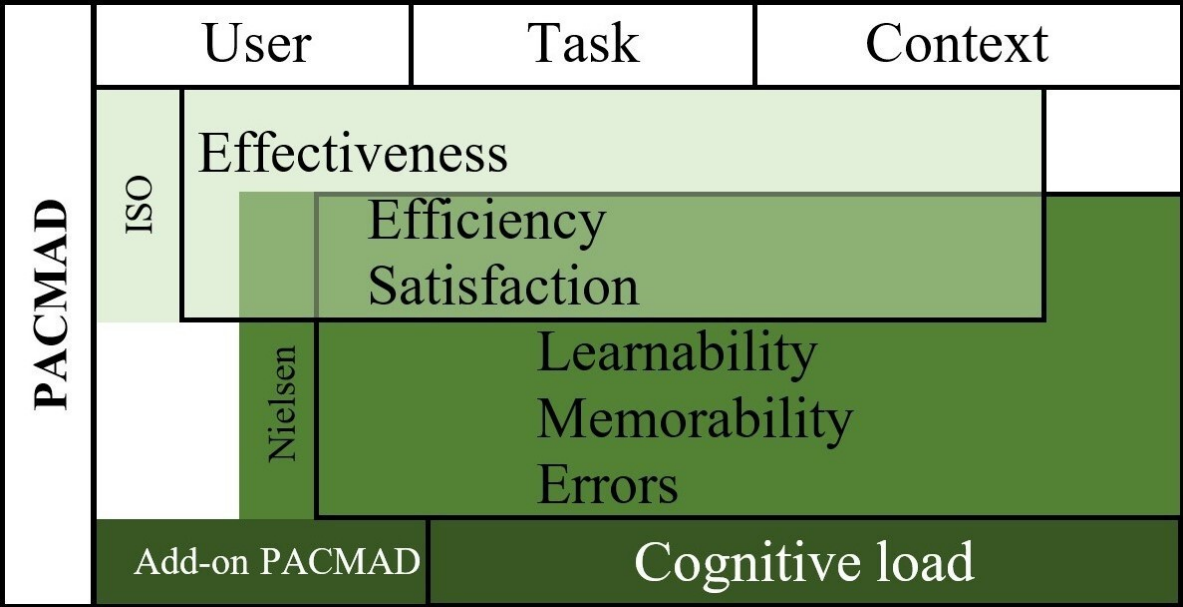
PACMAD usability model (own representation, based on a study by Harrison et al). ISO: International Organization for Standardization; PACMAD: People at the Centre of Mobile Application Development.

The evaluation of the DiGA is based on the PACMAD usability model (Figure 1 [22]), which combines usability models developed by the International Organization for Standardization [26] and Nielsen [27] with an additional focus on mobile applications [22].

The PACMAD usability model addresses 7 usability attributes in total: effectiveness, efficiency, satisfaction, learnability, memorability, errors, and cognitive load [22]. The latter attribute, as an additional component to existing usability models, is considered the main contribution of the PACMAD model. Within the scope of those attributes, 3 further factors can influence the overall usability: user (eg, previous experiences interfering with overall usability), task (eg, goals of users), and context of use (eg, environment of use) [22].

**Figure 2.**
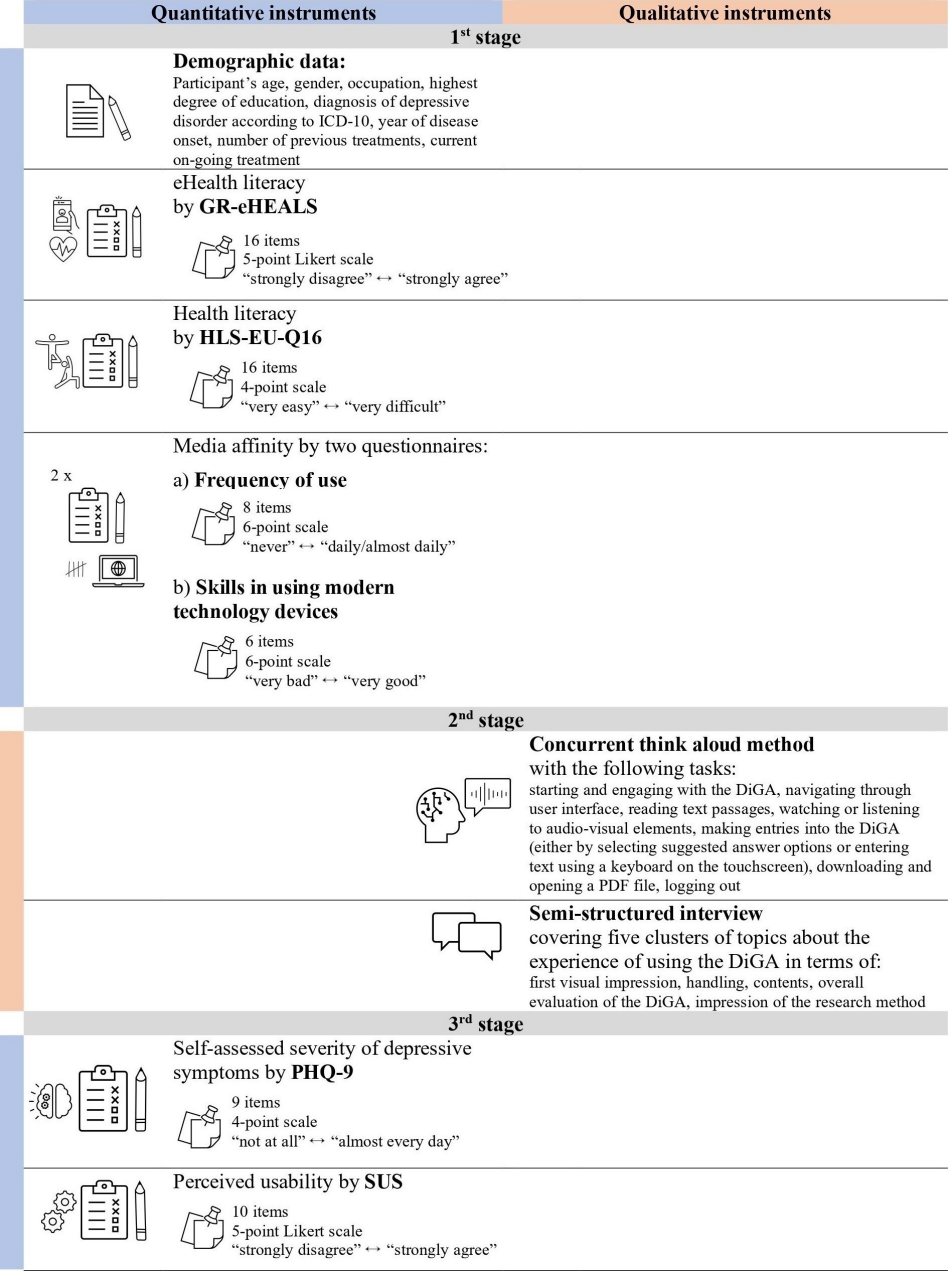
Data collection and instruments (own representation). DiGA: digital health applications; GR-eHEALS: revised German eHealth Literacy Scale; HLS-EU-Q16: European Health Literacy Survey Questionnaire; *ICD-10*: *International Statistical Classification of Diseases, Tenth Revision*; PHQ-9: 9-item Patient Health Questionnaire; SUS: System Usability Scale.

The data collection incorporated quantitative instruments in the first and third stages and qualitative instruments in the second stage (Figure 2) [28-32].

The revised German eHealth Literacy Scale (GR-eHEALS), the European Health Literacy Survey Questionnaire (HLS-EU-Q16), and the System Usability Scale (SUS) were used. During the third stage, a validated questionnaire was used for the self-assessment of the severity of depressive symptoms. The PHQ-9 is a widely used screening instrument for assessing depressive symptoms, based on *Diagnostic and Statistical Manual of Mental Disorders* (*DSM*) criteria. Its brevity, diagnostic utility, and ease of administration make it especially suitable for use in population-based studies, including those involving older adults. The German version of the PHQ-9 was developed through a rigorous process of translation and back-translation, ensuring cultural and linguistic equivalence. Validation studies conducted in Germany have confirmed the semantic and conceptual appropriateness of the questionnaire for older German speakers [31]. In the German older adult population, the PHQ-9 has shown good criterion validity, with sensitivity and specificity levels that are consistent with its performance in younger populations. For instance, Kocalevent et al [33] reported a sensitivity of 81% and specificity of 89% at the standard cutoff score (≥10) when compared with structured clinical interviews. On the basis of these findings, the PHQ-9 can be considered a valid and reliable instrument for assessing depressive symptoms in older adults in Germany.

The concurrent think-aloud method [34] is a common procedure in the field of digital health care for testing technologies in terms of usability, content, and accuracy of fit [27]. It enables researchers to trace the user’s course of action and reveals emerging usability issues [35] while offering insight into the users’ cognitive and emotional reactions and processes [36]. The tasks were based on typical activities during initial use. Furthermore, semistructured interviews were conducted to gather participants’ individual experiences and impressions after using the DiGA, enabling comparability while accommodating participants’ personal perspectives [37]. The second stage included audio recording as well as video capturing of the participant’s screen activity during DiGA use.

### Data Analysis

Quantitative data were analyzed by descriptive statistics using the statistical software package SPSS Statistics 27.0 (IBM Corp). Measures of central tendency were calculated for the comparison of individual items or sum scores between individual participants or between the groups. Reporting includes both the mean with SD and the median with IQR. To prevent case exclusion due to missing values, median imputation was applied to 2 individual responses within the eHEALS and SUS instruments.

The extraction and synthesis of qualitative data were carried out according to Rädiker and Kuckartz [38] using the software MAXQDA 2022 (VERBI Software GmbH). The predefined attributes of the PACMAD model were used as the main category system, supplemented by an additional category on methodology.

Initially, 2 researchers (MC and TSB) independently coded 1 Selfapy assessment and discussed the resulting variations and discrepancies to set a standard. The remaining 17 assessments were coded (MC), and uncertainties across all videos were discussed (MC and TSB).

To ensure interrater reliability, 4 assessments were reevaluated by 2 researchers (ICO and TSB), with each researcher rating 1 Selfapy and 1 deprexis assessment from 2 distinct hospital departments. In total, 5 of 18 assessments were viewed twice.

**Figure 3.**
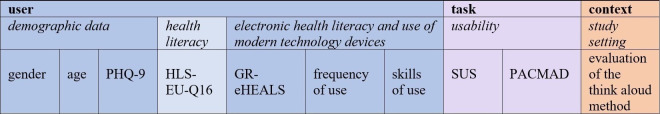
Triangulation matrix (own representation). GR-eHEALS: revised German eHealth Literacy Scale; HLS-EU-Q16: European Health Literacy Survey Questionnaire; PACMAD: People at the Centre of Mobile Application Development; PHQ-9: 9-item Patient Health Questionnaire; SUS: System Usability Scale.

The triangulation of both quantitative and qualitative datasets (Figure 3) [22,28-32] ensured a comprehensive interpretation of the PACMAD usability model. The factors user, task, and context of use are interconnected and compared across DiGA groups.

## Results

### Overview

A total of 18 evaluable assessments were conducted; 1 additional assessment could not be evaluated due to a technical malfunction of the recording equipment. Thus, each DiGA is represented by 9 assessments, equally covered by 3 per hospital department. All assessments were carried out with a study tablet, using the web-based application of both DiGA.

### Quantitative Findings

Quantitative data were analyzed for 18 participants aged 61 to 88 years, with an almost equal distribution in both groups. The participants’ average age was 68.3 (SD 9.6) years, and the majority (n=14, 78%) were women. Each group consisted of 7 women and 2 men. All but 3 participants were no longer employed (n=15, 83%).

On average, participants scored 12.4 (SD 5.2) on the PHQ-9, indicating moderate depression. Eight (44%) participants, 3 from the deprexis and 5 from the Selfapy group, were undergoing outpatient depression treatment at the time of data collection.

Similar results were observed in both groups for the HLS-EU-Q16, indicating moderate to good health literacy (scale 16‐64) and for the GR-eHEALS, which represents moderate electronic health literacy (scale 16‐80; Table 1). A total of 17 (94.4%) participants reported using a smartphone daily or almost daily. Although purposeful sampling was applied, participants in the Selfapy group showed lower media affinity overall, both in terms of usage frequency and self-assessed technology skills. The findings on media affinity are based on the assessment of individual items, as the questionnaires are not validated for a score.

**Table 1.** Patient-reported parameters.

	Deprexis (n=9)		Selfapy (n=9)		Total (N=18)	
	Mean (SD)	Median (IQR)	Mean (SD)	Median (IQR)	Mean (SD)	Median (IQR)
Age (years)	68.33 (10.11)	63.00 (62.00-77.00)	68.22 (9.64)	63.00 (62.00-76.50)	68.28 (9.58)	63.00 (62.00-74.25)
Depressive severity (PHQ-9^a^) [31]^b^	12.11 (4.59)	13.00 (8.50-15.50)	12.63 (6.19)	12.50 (6.50-16.50)	12.35 (5.23)	13.00 (8.00-15.50)
Health literacy (HLS-EU-Q16^c^) [29]	45.78 (5.65)	48.00 (42.00-50.00)	46.33 (8.79)	46.00 (38.00-54.50)	46.06 (7.17)	47.00 (41.25-50.00)
Electronic health literacy (GR-eHEALS^d^) [28]	51.89 (6.03)	52.00 (47.00-57.50)	48.75 (14.48)	47.00 (41.00-57.00)	50.41 (10.60)	49.00 (46.50-56.00)
System Usability Scale [32]	64.44 (20.03)	67.50 (48.75-82.50)	55.83 (18.29)	60.00 (41.25-68.75)	60.14 (19.13)	62.50 (43.125-75.00)

a PHQ: 9-item Patient Health Questionnaire.

b For PHQ-9, the sample size was 17 (n=8 for Selfapy) due to missing data from 1 participant.

c HLS-EU-Q16: European Health Literacy Survey Questionnaire.

d GR-eHEALS: revised German eHealth Literacy Scale.

**Figure 4.**
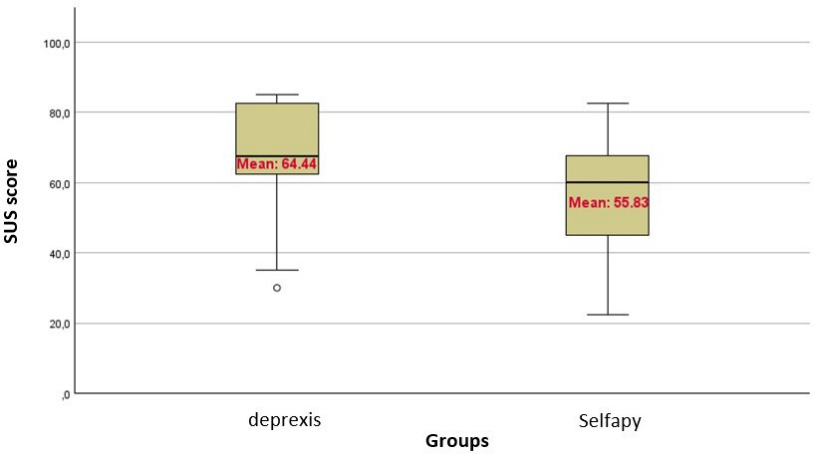
SUS score. SUS: System Usability Scale.

The usability assessment by SUS showed a higher level of user-friendliness for deprexis with an average score of 64.4 (SD 20.0) compared to Selfapy with 55.8 (SD 18.3). It is important to consider the SUS scores alongside participants’ individual skills, as they affect the DiGA’s perceived usability. Table 1 provides an overview of the self-reported patient parameters, and Figure 4 shows the SUS score distribution using a boxplot to visualize and compare the scatter, central tendency, and outliers across both groups.

### Qualitative Findings

#### Overview

Qualitative findings are presented using the main categories, combining the results for Selfapy and deprexis. The evaluation dimension of the categories differs: while effectiveness, efficiency, and errors were solely evaluated externally by the researcher, satisfaction represents a category that only the participant could self-assess. Learnability, memorability, cognitive load, and the handling of the think-aloud method could be assessed by the participant’s comments and through the researcher’s observation.

#### Methodology

Responses to the think-aloud method varied: Selfapy participants rated it either as not disturbing at all or as stressful. Positive comments in the deprexis group outweighed, although some users struggled with verbalizing their thoughts. A few participants requested that the texts be read aloud, finding it challenging to manage both the printed instructions and the digital device.

#### Learnability

The category learnability describes the ease with which proficiency can be achieved [22]. Participants in both groups were eager to enhance their proficiency and expressed a desire to gain more confidence. They acknowledged that while learning requires repetition, it was generally manageable. A few participants lacked basic technical understanding and asked, for instance, what a PDF file is or how to adjust the volume. Despite initial challenges, some skills were learned even during first-time use, such as scrolling, identifying the menu icon, opening the keyboard, and zooming. Navigation was found to be learnable, and repetition of certain tasks led to faster performance. Participants recommended more guidance for using DiGA, noting the study conductor’s help was valuable. They noted that, especially for older people without technical experience, the first use could be difficult.

#### Effectiveness, Efficiency, and Errors

The category effectiveness describes whether a task is completed by the user. Efficiency considers the speed and accuracy of completion, reflecting the user’s productivity. Furthermore, the PACMAD model proposes an evaluation of the errors made while using an application [22].

Most tasks were completed successfully, although often with the assistance of the study conductor. Both groups faced touchscreen issues, causing frustration (eg, having to tap multiple times). Both DiGA presented navigation challenges, including orienting within chapters, finding PDF files, returning to the main page or the previous section, and the logout process. Reading and comprehension issues stemmed from small font size or unclear instructions and questions within both DiGA.

In Selfapy, the chapter overview layout led to confusion, with participants expecting immediate access to the chapter upon clicking. Other challenges included scrolling, selecting between answer options, and entering text via the touchscreen keyboard. Owing to Selfapy’s user interface design, participants often missed the *next* button after completing 1 specific task and were confused about how to proceed. The required choice between 2 video formats (web based or app based) caused uncertainty about which video to watch. Selecting the wrong format resulted in limited visibility. Two participants in the Selfapy group terminated the assessment before completing all the tasks.

Deprexis users encountered similar but fewer issues. Specifically, some users failed to open a PDF file, as they clicked on the adjacent nonresponsive description instead of the PDF icon. They additionally faced difficulties locating the menu, as they were not familiar with the menu symbol. In some cases, the audio file was either overlooked or not played, despite having been noticed.

#### Satisfaction

The user’s attitudes toward the DiGA provided insight into their satisfaction with using it [22]. Difficulties with navigation and the handling of the DiGA were recognized by the participants. One-third of the Selfapy users found the menu navigation and overall user interface pleasant, well structured, and intuitive, whereas the majority criticized the navigation as unintuitive and difficult to understand, requiring assistance in the beginning. Some participants suggested that repeated use could lead to improvement. One participant showed clear signs of frustration due to navigation difficulties.

In contrast, two-thirds of deprexis users perceived the DiGA as well structured and easy to navigate, whereas the remaining third found the navigation to be difficult and unintuitive. The main page and the beginning of the assessment were perceived as positive and accessible. Suggestions included clearer menu labels and a single-column layout of the menu to improve navigation.

Regarding design and layout, participants liked Selfapy’s appearance, although some found the font size too small and the color contrast insufficient, restricting visibility. Deprexis was well received for its simple, clear interface, although opinions varied on visual elements and color use. The presentation of information was praised as well arranged, but participants recommended increasing the PDF font size. With regard to the general font size, while no negative feedback was given, 2 participants noted it was sufficiently large.

Content and the functions of both DiGA were generally perceived positively. The information was found to be presented in accessible language, with adequate depth and structure. The content was described as interesting, informative, and helpful; however, participants criticized the text in both DiGA as overly lengthy. They suggested an audio playback feature to resolve this concern. Some participants found the answer options limiting, whereas others praised the possibility of individual choice. Besides Selfapy’s diary function and support options, the video was praised for its content, graphics, and brevity, although participants suggested moderating the voice-over pitch and reducing the pace.

Deprexis users appreciated the mood check, despite some criticism of its retrospective nature, and expressed a desire for additional support during completion. The overall guidance and motivation provided by deprexis, along with the relaxation exercises and the required responses, were well received. The audio element was praised for being engaging, clear, and memorable, with a pleasant voice quality and appropriate length.

The adoption of Selfapy for long-term use was met with mixed reactions. Some users were deterred by technical constraints and uncertainty about the effectiveness of managing illness. Others showed readiness to use DiGA, encouraged by its interactive nature and constructive suggestions. However, reluctance persisted due to general skepticism or insufficient technical equipment. Overall, the sentiment toward Selfapy was cautious yet open to possibilities, with a clear need for support and reliable information.

In contrast, deprexis users approved of the DiGA, finding it a reliable aid. Many participants were open to using it regularly, particularly during critical health periods, and valued its potential at the onset of the disease to bridge therapy waiting times. Participants saw deprexis as a valuable addition to therapy but were unsure about its usefulness for individuals with severe depression. Some preferred personal interaction over the digital interface, whereas others liked the independence it offers. Concerns were raised regarding the data security of the diary feature, despite its perceived benefits. Doubts persisted regarding DiGA’s capacity to meet individual needs and its potential to replace consultations with a therapist. A common concern was that using the DiGA alone could be overwhelming, prompting suggestions for an introductory course.

#### Cognitive Load

Cognitive load addresses the extent of cognitive processing while using an application [22]. Difficulties were prominent in this study: participants struggled with text comprehension and production, felt overwhelmed and insecure, and had memory and concentration lapses. These difficulties impacted their self-assessment. Both DiGA user groups faced these issues, but Selfapy was perceived as particularly challenging.

Selfapy users often struggled to grasp certain terms and instructions within the DiGA, frequently asking additional questions for clarity. They felt overwhelmed by the complexity and amount of content, struggled to articulate their thoughts in written form, were concerned about grammatical or spelling errors, and noted a decline in ability associated with age. Some Selfapy users stated that they had reached their individual limit at times and voiced uncertainty about their ability to concentrate and retain information after a single reading. Selfapy users experienced a powerful sense of self-doubt and insecurity. Despite their efforts, they harshly attributed mistakes to their own reduced intelligence and technical skills, rather than considering external factors or task complexity.

Deprexis users preferred shorter texts than those provided, despite understanding the content, and encountered minor comprehension difficulties with some tasks and instructions within the study. No serious confusion during the usage of deprexis was observed. Challenges included reduced focus due to illness, racing thoughts, and a reliance on routines to manage depression. Participants also expressed concern about self-esteem and mood assessment, which both influence cognitive processing.

#### Memorability

Memorability is defined as the user’s ability to retain how an application is used effectively [22]. A second assessment is typically needed to evaluate memorability, but initial difficulties were evident during first-time use. From the participants’ perspective, the video in Selfapy contained an excessive amount of information, with participants unable to remember important instructions, such as how to log off. Some did not recall watching a video at all when asked. Deprexis users also showed a few signs of limited memorability: 1 participant filled out the mood check twice without realizing it, and 2 participants forgot about the audio element when asked about it. A reminder feature was proposed for consistent DiGA use.

## Discussion

### Principal Findings

This prospective observational study gives new insights into the usability of DiGA for older adults with regard to depression, particularly focusing on deprexis and Selfapy, which were the first 2 permanently approved DiGA for depressive disorders. By focusing on participants aged ≥60 years, this research addresses the disparity in usage among older individuals, who have not been adequately represented in previous research. The study advocates for a user-centered approach in DiGA development on the basis of the PACMAD model. The study used a mixed methods approach. While the user groups were comparable in age, gender, severity of depressive symptoms, and health literacy, Selfapy users reported slightly lower technical proficiency and usability scores. Design flaws and increased cognitive load were reported by the participants of the Selfapy group. Despite visual, motor, and cognitive challenges, participants in both groups emphasized the need for support and practice in the use of DiGA for older adults.

A study by Wildenbos et al [39] on barriers to digital health among older adults identified 4 main categories of age-related barriers that can affect the usability of mobile health: physical abilities, perception, cognition, and motivation. Similar findings emerged from the present qualitative study results, corroborating the slightly lower SUS score for Selfapy compared to deprexis.

### Barriers to Physical Abilities

Visual and psychomotor limitations were observed universally in both groups and were also evident in the participants’ criticism regarding difficulties in using the touchscreen and navigation. Wildenbos et al [39] also reported that other studies had found slowed movements, reflexes, and tremors to be a challenge. It is assumed that these physical limitations may also affect learning duration, speed of performance, error rate, time retention, and subjective satisfaction.

### Barriers to Perception

Barriers of perception were reflected in the early termination of 2 Selfapy assessments, increased error rates due to design limitations, and more moments of confusion and uncertainty with navigation, indicating usability constraints. In addition, small font size and poor color contrast were criticized. Wildenbos et al. [39] highlight the need for enhanced lighting to support visual clarity, as well as implications for video content and alerts due to the age-related decline in hearing ability. Hearing difficulties may also result in lower use of desktop computers and internet services compared to older adults without hearing impairments [40].

### Barriers to Cognition

Selfapy users also experienced pronounced cognitive load. Some participants showed reduced concentration and struggled with text comprehension. This area may be less prominent in the present study compared to the study by Wildenbos et al. [39], identifying potential challenges because this study did not include individuals with cognitive impairments. However, the participants’ illnesses must be considered as a contributing factor.

### Barriers to Motivation

Motivation is one of the key factors influencing the acceptance of technology [41]. Acceptance theories can provide exciting insights in this regard. Although the present study initially focused on usability, tentative conclusions can be drawn regarding this barrier: Selfapy users were more self-critical when faced with challenges. A reason for this could be the lower level of proficiency with modern technology reported by Selfapy users. Here, the mixed methods approach proved to be helpful in capturing specific impressions during use, in addition to the SUS.

Help with increasing motivation can be found in the literature: a systematic review examined the extent to which characteristics of the design and implementation of digital interventions can increase adherence among vulnerable population groups (older people, people with low socioeconomic status, single parents, social minorities, and people with a migration background). The authors state that multimodal content and direct opportunities for interaction and support between users and intervention providers can increase usage [42].

### Interventions for Successful Implementation

Although participants encountered difficulties, they were mostly satisfied with both DiGA and expressed encouragement for long-term use. Successful long-term use depends particularly on actual use in everyday life. It is important to note that digital psychological treatment may have higher dropout rates compared to analog options [43]. A key factor in this area is adherence. A systematic review of adherence to digital interventions for mental health conditions found that female gender, positive treatment expectations, and the availability of personal support can increase adherence. Negative factors include limited time for engagement, personal dissatisfaction with the intervention content, or content perceived as impersonal [44]. The positive assessment of personal support was also evident in this study because both user groups rated their assigned DiGA as suitable for older people, emphasizing the necessity to practice the usage.

Although most of the tasks were successfully completed, participants received light support from the study conductors during use to reduce psychological strain. In traditional postprescription use, such support would not be available. The authors, therefore, expect participants to have more difficulty coping with the application, despite its approval through the DiGA authorization procedure. Structured support could potentially be helpful in this context. For example, it became apparent that a structured support system using weekly brief semistandardized emails achieved a high level of effectiveness for a digital mental health application [45]. Other studies on the use of mobile apps for mental health among older users also support the value of this approach [46-48].

Moreover, explicit guidelines and recommendations for developers could be beneficial in mitigating usability-related challenges. While such recommendations should not be derived solely from pilot studies focusing on usability, such as this study, there is a substantial body of design research that provides well-founded guidance in this area. For instance, Gomez-Hernandez et al [49] conducted a systematic review and thematic analysis on design guidelines for mobile applications targeted at older adults. Their study identified a total of 27 key recommendations, including 2 “golden rules” and 25 design principles, which were organized into 5 thematic categories: support and training, navigation, visual design, cognitive load, and interaction.

### Strengths and Limitations

The distributions must be interpreted with caution due to the small sample size: it increases the variability of the data and limits the statistical validity of the results. In particular, outliers can distort the overall picture and thus impair the reliability of the conclusions derived.

This study gives new insights into the usability of DiGA for older adults with regard to depression, particularly focusing on deprexis and Selfapy, which were the first 2 permanently approved DiGA for depressive disorders. By focusing on participants aged ≥60 years, this research addresses the disparity in usage among older individuals, who have not been adequately represented in previous research. The study advocates for a user-centered approach in DiGA development on the basis of the People at the Centre of Mobile Application Development usability model. The distributions must be interpreted with caution due to the small sample size: it increases the variability of the data and limits the statistical validity of the results. In particular, outliers can distort the overall picture and thus impair the reliability of the conclusions derived.

On the basis of the information assessed by the GR-eHEALS and the 2 questionnaires on media affinity, the participants in the Selfapy group showed slightly lower proficiency in using modern technology than those in the deprexis group, which could have influenced the usability assessment.

Although the process of actively verbalizing one’s thoughts is assumed to be unfamiliar, the participants were largely unaffected by the methodology. However, it must be taken into consideration that 3 different study conductors (1 per hospital department) were involved in data collection. Although the study settings through uniform training of the involved study members, minor procedural differences were nonetheless observed.

Another limitation arises from the target group of the study. Depressive disorder is characterized by symptoms such as attention deficits and lack of motivation. This may have impacted the perceived ease of use, although this effect cannot be conclusively demonstrated. However, we considered it crucial to focus on this target group to better understand the challenges faced by this underrepresented group. It is also important to note that participants were recruited directly from clinical practice. This approach was criticized in a meta-study on the effectiveness of deprexis, as the participants in those studies were recruited outside the clinical context, potentially compromising the transferability of the results [50].

### Further Research

Larger controlled studies will be needed to replicate the findings and explore further moderators of usage outcomes. While the PACMAD usability model was designed specifically for assessing the usability of mobile applications and takes cognitive load into account, other factors influencing general technology acceptance are not considered.

The subsequent study of the DiGA4Aged project, which is the pilot study, will also examine technology acceptance and the success of support provided to participants by a digital nurse within an RCT. In the future, studies incorporating an acceptance model such as the Unified Theory of Acceptance and Use of Technology [51], which focuses more on general technology acceptance and less on mobile-specific use, will provide valuable insights. Furthermore, research on the implementation of DiGA for this specific patient group could be undertaken, possibly considering the Consolidated Framework for Implementation Research [52].

### Conclusions

The findings of this study highlight the need to enhance the accessibility and usability of DiGA for older adults with depressive disorders, who face unique challenges due to age-related sensory, motor, and cognitive changes. In addition, the results show that usability was perceived as lower in the Selfapy group, primarily due to design-related factors of the application. Users in this group reported experiencing greater cognitive load. Participants in both groups emphasized the importance of receiving support when using the DiGA and expressed their desire to continue using it. However, further research is required to identify potential support measures for the use of DiGA by older adults, as well as factors influencing adherence. This way, DiGA can become more accessible and beneficial, ultimately reducing health care disparities and enhancing the quality of life for this vulnerable group. There is a necessity to take into account the needs of the target groups of DiGA in the authorization process and increase efforts to offer older adults support to bridge the digital divide. Considering the study’s results and limitations, the decision of the DiGA to be used in the upcoming RCT was made in favor of deprexis.

## Abbreviations

DiGA: digital health applications
*DSM*: *Diagnostic and Statistical Manual of Mental Disorders*
GR-eHEALS: revised German eHealth Literacy Scale
GRAMMS: Good Reporting of a Mixed Methods Study
HLS-EU-Q16: European Health Literacy Survey Questionnaire
PACMAD: People at the Centre of Mobile Application Development
PHQ-9: 9-item Patient Health Questionnaire
RCT: randomized controlled trial
STROBE: Strengthening the Reporting of Observational Studies in Epidemiology
SUS: System Usability Scale
